# A machine learning framework develops a DNA replication stress model for predicting clinical outcomes and therapeutic vulnerability in primary prostate cancer

**DOI:** 10.1186/s12967-023-03872-7

**Published:** 2023-01-12

**Authors:** Rong-Hua Huang, Ying-Kai Hong, Heng Du, Wei-Qi Ke, Bing-Biao Lin, Ya-Lan Li

**Affiliations:** 1 Department of Anesthesiology, The First Affiliated Hospital of Jinan University, Guangzhou, 510630 Guangdong China; 2 Department of Urology, The First Affiliated Hospital of Shantou University Medical College, Shantou, 515000 Guangdong China; 3 Department of Secretion, Baoji Central Hospital, Baoji, 721008 Shaanxi China; 4 Department of Anesthesiology, The First Affiliated Hospital of Shantou University Medical College, Shantou, 515000 Guangdong China; 5 Department of Urology, Kidney and Urology Center, Pelvic Floor Disorders Center, The Seventh Affiliated Hospital, Sun Yat-Sen University, Shenzhen, 518000 Guangdong China

**Keywords:** Machine learning, Prostate cancer, DNA replication stress, Precision oncology

## Abstract

Recent studies have identified DNA replication stress as an important feature of advanced prostate cancer (PCa). The identification of biomarkers for DNA replication stress could therefore facilitate risk stratification and help inform treatment options for PCa. Here, we designed a robust machine learning-based framework to comprehensively explore the impact of DNA replication stress on prognosis and treatment in 5 PCa bulk transcriptomic cohorts with a total of 905 patients. Bootstrap resampling-based univariate Cox regression and Boruta algorithm were applied to select a subset of DNA replication stress genes that were more clinically relevant. Next, we benchmarked 7 survival-related machine-learning algorithms for PCa recurrence using nested cross-validation. Multi-omic and drug sensitivity data were also utilized to characterize PCa with various DNA replication stress. We found that the hyperparameter-tuned eXtreme Gradient Boosting model outperformed other tuned models and was therefore used to establish a robust replication stress signature (RSS). RSS demonstrated superior performance over most clinical features and other PCa signatures in predicting PCa recurrence across cohorts. Lower RSS was characterized by enriched metabolism pathways, high androgen activity, and a favorable prognosis. In contrast, higher RSS was significantly associated with TP53, RB1, and PTEN deletion, exhibited increased proliferation and DNA replication stress, and was more immune-suppressive with a higher chance of immunotherapy response. In silico screening identified 13 potential targets (e.g. TOP2A, CDK9, and RRM2) from 2249 druggable targets, and 2 therapeutic agents (irinotecan and topotecan) for RSS-high patients. Additionally, RSS-high patients were more responsive to taxane-based chemotherapy and Poly (ADP-ribose) polymerase inhibitors, whereas RSS-low patients were more sensitive to androgen deprivation therapy. In conclusion, a robust machine-learning framework was used to reveal the great potential of RSS for personalized risk stratification and therapeutic implications in PCa.

**Supplementary Information:**

The online version contains supplementary material available at 10.1186/s12967-023-03872-7.

## Introduction

Prostate cancer (PCa) was the second most commonly diagnosed cancer and the fifth leading cause of cancer death in 2020 [1]. Common treatment options for PCa include active surveillance, surgery, androgen deprivation therapy (ADT), taxane chemotherapy, and Poly (ADP-ribose) polymerase (PARP) inhibitors [2]. Because PCa is considerably heterogeneous between patients and even within patients, treatment modalities must be tailored to account for genomic and clinical differences between patients [3, 4]. However, current evidence suggests that clinical characteristics and existing tests such as Gleason scores, serum prostate-specific antigen (PSA), and BRCA1/2 mutations are insufficient to predict PCa progression or guide treatment regimens [5–7]. As a result, patients with PCa are at great risk of being over-treated or under-treated [8], and many of these patients will eventually develop metastatic PCa [9, 10]. With the advancement of high throughput sequencing, there has been great interest in harnessing this technology to narrow the gap in PCa personalized medicine [11–13].

Replication is a sophisticated process in which DNA is faithfully duplicated [14]. Any impediment to this highly regulated process is known as DNA replication stress which can lead to genomic instability and, in the long run, promote tumorigenesis and progression [14]. The DNA damage response (DDR) is required to maintain genome integrity by repairing DNA damages and restoring proliferation, or by inducing cellular senescence or apoptosis [15]. Dreyer et al. found that DNA replication stress existed independently of DDR in pancreatic cancer and opened up different therapeutic opportunities [16]. Nyquist et al. reported that combined RB1 and TP53 loss accelerated cell proliferation and exhibited replication stress, was significantly associated with poor survival, and conferred sensitivity to combined PARP and ATR inhibitors in metastatic PCa [17]. Therefore, it’s necessary to assess the levels of replication stress in PCa patients for precise risk stratification and treatment guidance. However, patients with primary PCa may exhibit varying degrees of replication stress in the absence of genetic alterations, which hinders the early identification of those with higher replication stress. Genomic and epigenomic changes often act by regulating mRNA expression. Given that transcriptomic signature for replication stress in PCa have not been investigated, we herein attempt to quantify DNA replication stress at the transcriptomic level in primary PCa.

This study established a robust replication stress signature (RSS) by benchmarking 7 survival algorithms and showed that RSS had better predictive power than common clinical variables and other PCa signatures in multiple independent cohorts. In addition, in silico screening identified 13 potential therapeutic targets and 2 therapeutic agents for RSS-high patients. Furthermore, we showed that the knockdown of FEN1 and RFC5 could inhibit cell growth and promote apoptosis in PCa cell lines.

## Materials and methods

### Data collection and processing

Somatic mutation and copy number alteration data were retrieved from The Cancer Genome Atlas (TCGA) database (http://cancergenome.nih.gov/). mRNA expression data and corresponding clinical information of the TCGA-PRAD cohort (n = 488) were downloaded from XENA (https://xena.ucsc.edu/) in November 2020. A total of 4 external validating datasets including GSE70769 (n = 92), GSE70768 (n = 111), GSE94767 (n = 132), and DKFZ-PRAD (n = 82) were collected from the Gene Expression Omnibus (GEO, https://www.ncbi.nlm.nih.gov/geo/) and the cBioPortal (https://www.cbioportal.org/). We only include primary PCa samples with available survival data. For somatic mutation data, we followed the TCGA MuTect2 pipeline and used “maftools” package to perform downstream analysis. GISTIC2.0 (Gene Pattern) was leveraged to detect somatic copy number variations [18]. The predicted aneuploidy score, tumor mutation burden, and tumor neoantigens data were retrieved from Thorsson et al. [19]. The HRDetect-score measures the degree of homologous repair deficiency in PCa using genomic data and was derived from a previous study by Sztupinszki et al. [5]. For RNA sequencing data, we used log2(transcripts per million + 1) as the expression levels of mRNA while for raw expression data from microarrays, we performed background adjustment and normalization using the robust multiarray average algorithm (‘affy’ R package). Since batch effect exists across cohorts, we applied ‘combat’ function in the ‘sva’ R package to remove the batch effect and merge them into a Meta-cohort (Additional file 2: Figure S1A, B). Disease-free survival and biochemical recurrence survival are both considered primary endpoints.

In addition, we retrieved clinical information and RNA sequencing data from the IMvigor210 cohort, which evaluated the efficacy of atezolizumab, an anti-programmed death-ligand 1 inhibitor in urothelial carcinoma [20]. RNA sequencing data of human cancer cell lines and corresponding drug sensitivity data were curated from the Cancer Cell Line Encyclopedia project (https://portals.broadinstitute.org/ccle/) and the secondary PRISM Repurposing dataset (19Q4, https://depmap.org/portal/prism/), respectively. A total of 1448 compounds were screened against 489 cancer cell lines. The drug response was measured by area under the dose–response curve (AUC) values with lower values indicative of higher sensitivity. We also downloaded CERES scores for 17,386 genes in 1086 cancer cell lines from the dependency map (22Q2, https://depmap.org/portal/). CERES was used to estimate gene-dependency levels from CRISPR-Cas9 essentiality screens. A lower CERES score indicates the gene of interest is more likely to be essential in a particular cancer cell line.

### Machine learning benchmark for construction of DNA replication stress signature

We curated 21 DNA replication stress signatures from a previous study [16] (Additional file 1: Table S1) and removed those that were not recovered in the PCa bulk datasets. We then performed univariate Cox regression analysis in the training cohort TCGA-PRAD to identify genes that were significantly associated with PCa biochemical recurrence (P < 0.01). To ensure the robustness of this selection, we adopted a bootstrap approach by sampling 80% of patients 1000 times and only retrieved genes with P < 0.01 more than 800 times. Furthermore, we used the Boruta algorithm with ntree = 1000 and maxRuns = 1000 to retain genes that were considered more relevant to clinical prognosis after comparing the importance of selected features and random features.

To establish an accurate and robust DNA replication stress signature (RSS), 7 machine learning algorithms including lasso, elastic network (Enet), Ridge, partial least squares regression for Cox (plsRcox), CoxBoost, eXtreme Gradient Boosting survival (XGBoost), and supervised principal components (SuperPC) were benchmarked via nested cross-validation (CV) in the TCGA-PRAD cohort [21–25]. Specifically, we tuned the hyper-parameters of each algorithm using the inner fivefold CV and evaluated the performance of the best-tuned models using the outer tenfold CV. We maintained the same ratio of non-recurrent and recurrent cases in each fold. The hyperparameters used for each algorithm were provided in Additional file 1: Table S2. A comprehensive performance evaluation was conducted by comparing the averages of the Harrell’s concordance index (C-index), integrated brier score (IBS), and 1-, 3-, 5-, and 10-year area under the ROC curve (AUC) values from 10 testing folds. The model with the highest average metrics was considered optimal. The function “xgb.importance” in the R package “xgboost” was utilized to help interpret the contribution of each feature to the model prediction.

### Comparison of RSS with published signatures

We retrospectively curated 3 PCa signatures for comparison, including Xu et al.’s 33 5-methylcytosine-related gene signature [26], Feng et al.’s 2 senescence-related gene signature [27], and Liu et al.’s 7 ferroptosis-related gene signature (Additional file 1: Table S3) [28]. The scaled normalized expression of genes was multiplied by the corresponding coefficients and then summed to yield the score for each sample. We then computed C-index and AUC values to compare the predictive powers of each signature.

### Differential expression analysis

Differential analysis was conducted between RSS-high and RSS-low groups using R package ‘Limma’. Differentially expressed genes were defined using adjusted P value < 0.05 and absolute log2(Fold-change) > 1. To obtain consensus gene expression patterns across bulk datasets, we further performed meta-analysis using a random effect model.

### Single-sample gene set enrichment analysis and immune cell infiltration

To assess the association of RSS with biological pathway activity, we leveraged single-sample gene set enrichment analysis (ssGSEA) to compute activity scores for the Hallmark gene sets and Kyoto Encyclopedia of Genes and Genomes gene sets (h.all.v7.4.symbols and c2.cp.kegg.v7.4.symbols, https://www.gsea-msigdb.org/gsea/msigdb/; Additional file 1: Table S4) in the Meta-cohort through ‘GSVA’ R package. To explore the impact of RSS on the tumor microenvironment, the CIBERSORT algorithm was adopted to quantify the relative fraction of 22 tumor-infiltrating immune cells in the Meta-cohort.

### In silico prediction of therapeutic targets and agents

As a large number of proteins lack binding sites or adequate affinity to small molecules or antibodies, we firstly collected 2249 druggable targets (Additional file 1: Table S5) from a previous publication [29]. We then screened for targets that were positively correlated with RSS scores using Spearman’s rank-order correlation analysis with correlation coefficients > 0.3 and false discovery rate < 0.05 as the thresholds. Lastly, we filtered out targets that had CERES scores > -1 in more than 1 PCa cell line.

To discover possible therapeutic agents, we firstly utilized connectivity MAP (CMap, https://clue.io/query), which compares a differential gene signature to all perturbational signatures in CMap and outputs a score measuring similarity between signatures [30]. Therefore, drugs with scores < -95 are considered candidates for reversing the RSS differential signatures. We also applied Ridge regression (R package “pRRophetic”) with tenfold CV to train a drug sensitivity prediction model by using the transcriptomic profiles and drug sensitivity data in PRISM. The fitted model was then used to predict AUC values of each drug for every PCa sample based on their transcriptomic profiles.

### Cell culture and transfection

We cultured the C4-2B and PC-3 cell lines using RPMI 1640 medium supplemented with 10% fetal bovine serum (FBS, Biological Industries, HAMEK, Israel) and 1% penicillin and streptomycin (100 U/mL, Biological Industries, HAEMEK, Israel). All cells were cultured at 37 °C and 5% CO_2_.

For gene silencing by RNA interference, siRNAs were synthesized by RiboBio (Guangzhou, China) and transiently transfected into cells using the Lipofectamine RNAiMAX reagent based on the manufacturer’s instructions. Cells were harvested after transfection for 2–3 days and used for further experiments. The siRNA sequences were 5’-AGAAUGACAUCAAGAGCUA-3’ for siFEN1 and 5’-GAUUCUGCCUCAUCUGUAA-3’ for siRFC5.

### Cell viability analysis and Colony formation assay

Transfected C4-2B and PC-3 cells were seeded into 96-well plates at 3 × 10^3^ cells/well. Cell viability was measured using the Cell Counting Kit-8 (CCK-8) assay (40203ES76, Yeasen Biotechnology, Shanghai, China) as per the manufacturer’s instructions. Cell viability was quantified by measuring the absorbance at 450 nm through a microplate reader (Model Synergy HTX, BioTek, Winooski, VT, USA) at 0, 24, 48, 72, and 96 h.

For colony formation assay, transfected cells were plated in 6-well plates (1000 cells per well). Cells were cultured for 10 or more days, fixed in 4% paraformaldehyde for 20 min, and stained with 0.25% crystal violet for another 20 min. The number of colonies was counted by the ImageJ program.

### Apoptosis assay

Transfected cells were harvested and stained with Annexin V (AV)-FITC and propidium iodide (PI) using the AV-FITC/PI Apoptosis Detection Kit (40302ES50, Yeasen Biotechnology, Shanghai, China). Apoptotic cells were counted according to AV and PI signals detected by flow cytometry (Attune NXT Acoustic Focusing Cytometer-AFC2, Invitrogen).

### RNA extraction and real-time qPCR

Total RNA was extracted by Eastep Super Total RNA Extraction Kit (Promega), and cDNA was synthesized with the HiScript III All-in-one RT SuperMix (Vazyme, Nanjing, China) using 1 ug RNA as input. Real-time qPCR was performed with the 2 × SYBR Green qPCR Mix (KT Life, Shenzhen, China). GAPDH was used as an internal control. Primer sequences were as follows: 5’-GTCTCCTCTGACTTCAACAGCG-3’ (GAPDH forward), 5’-ACCACCCTGTTGCTGTAGCCAA-3’ (GAPDH reverse), 5’-AGCCCGTGTATGTCTTTG-3’ (FEN1 forward), 5’-AGTCAGGTGTCGCATTAG-3’ (FEN1 reverse), 5’-GAAGCAGACGCCATGACTCAG-3’ (RFC5 forward), 5’-GACCGAACCGAAACCTCGT-3’ (RFC5 reverse).

### Western blotting

Transfected cells were rinsed with ice-cold PBS and lysed with buffer containing protease and phosphatase inhibitors. Protein samples were separated by 10% polyacrylamide gel electrophoresis and then transferred onto 0.45 μm pore-size polyvinylidene difluoride membranes*.* The membrane was blocked by 5% skimmed milk for 1 h, immersed with primary antibodies at 4 °C overnight, washed three times, and incubated with horseradish peroxidase-conjugated secondary antibody (1:4000) for 1 h at room temperature. Protein bands were then detected using ECL substrate reagents through an automatic chemiluminescence imager (ChampChemi, Beijing, China). Primary antibodies applied in this study include antibodies against FEN1 (1:1000, PTM Biolabs Inc., Hangzhou, China, cat# PTM-6960), RFC5 (1:1000, Absin Bioscience Inc., Shanghai, China, cat# abs118292), and GAPDH (1:2000, Cell Signaling Technology, Beverly, MA, USA, cat# CST5174S).

### Statistical analyses

All statistical tests were performed using R statistical software (v4.1.2) with a two-sided P value < 0.05 considered statistically significant. For comparison of continuous variables between two groups, Student’s t-test was used if the variables are normally distributed; otherwise, the Wilcoxon rank sum test was used. For comparison of categorical variables, Fisher’s exact or chi-square test was used dependent on sample sizes. The correlation between two continuous variables was analyzed using Spearman’s rank-order correlation. The Hazard ratio (HR) with a 95% confidence interval (CI) was calculated using Cox regression analysis (“Survival” R package). Survival analysis was performed by Kaplan–Meier analysis and a P value was derived from the log-rank test. Time-dependent receiver operating characteristic curve (ROC) analysis is carried out using the “survivalROC” R package, which was also used to determine the best cut-off for risk stratification. C-index was computed using the “Hmisc” R package and the comparison of C-indexes between groups was analyzed by “compareC” R package. IBS was calculated through “survcomp” R package.

## Results

### Study design overview

**Fig. 1:**
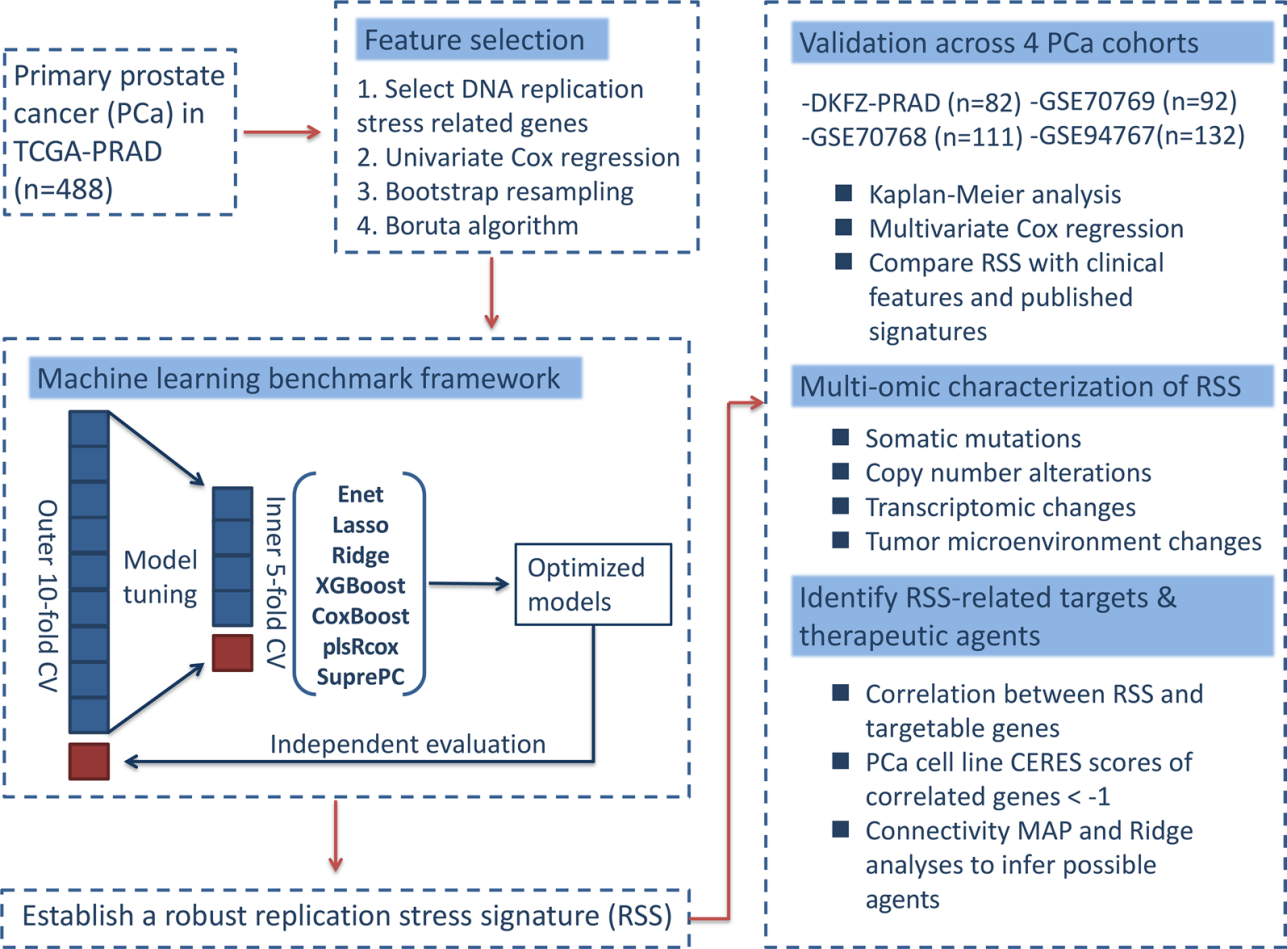
The workflow of the present study. (1) Feature selection and machine-learning benchmark were performed in the TCGA-PRAD dataset. (2) A replication stress signature was established and externally validated in 4 independent cohorts. (3) Potential therapeutic targets and drugs were identified through in silico screening

A total of 905 primary PCa cases with available survival data were included for downstream analysis. The flowchart of this study was shown in Fig. 1.

### Identification of DNA replication stress-associated features in TCGA-PRAD

**Fig. 2:**
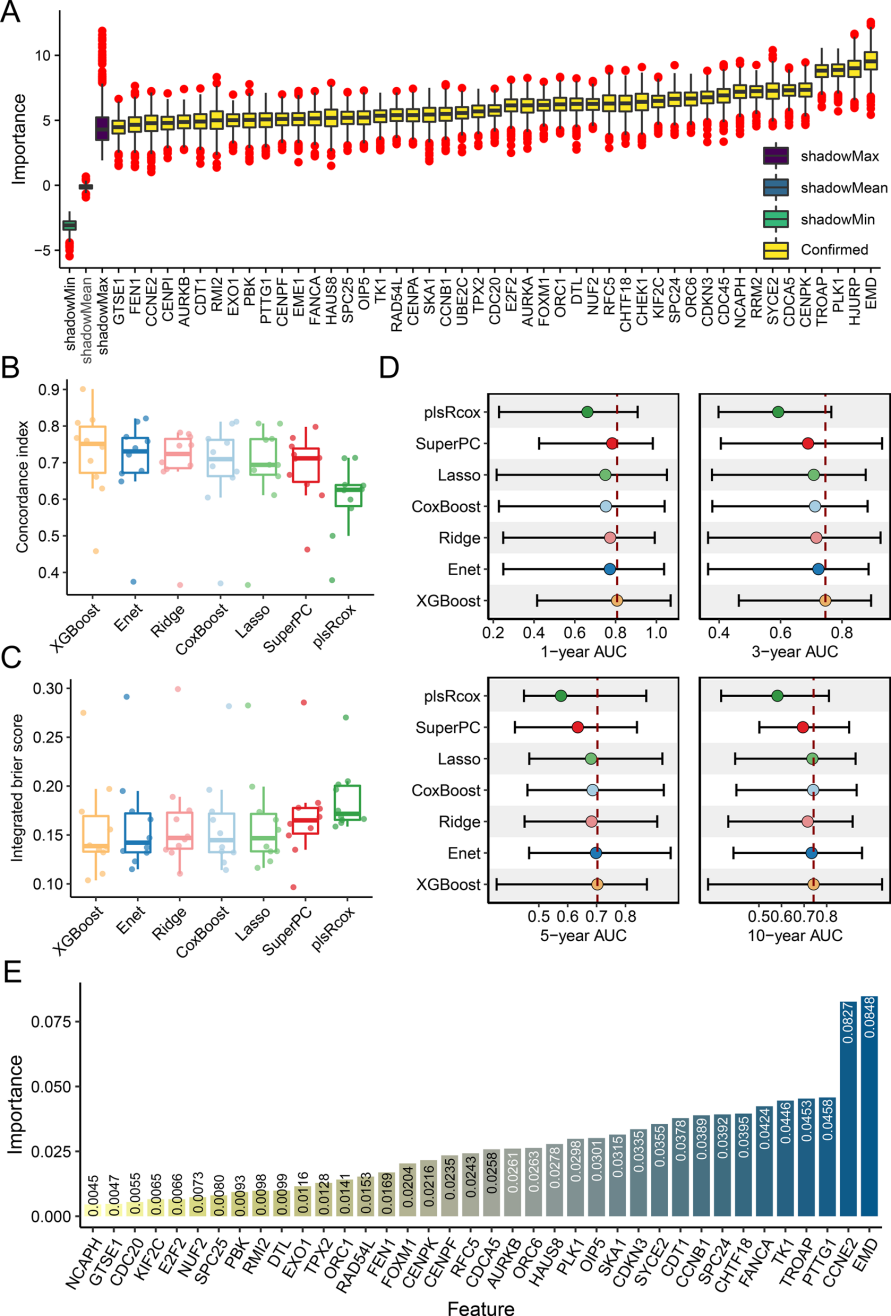
A robust replication stress signature (RSS) was developed by machine learning benchmark. **A** The Boruta algorithm identified 47 replication stress-related genes that were associated with PCa recurrence. Yellow represents confirmed features while other colors denote shadow attributes. The corresponding boxplots compared the concordance index (C-index) values (**B**) and integrated brier score (IBS) (**C**) of 7 survival-related machine learning algorithms using nest cross-validation. The individual dots correspond to the results of each independent validation. **D** Comparison of time-dependent area under the receiver operating characteristic curve (AUC) values at 1-, 3-, 5-, and 10-year among the machine learning algorithms. Dots indicate the average AUC values. **E** Bar plot of feature importance. Contributions of included genes for prostate cancer recurrence to the XGBoost model in the TCGA-PRAD cohort

A total of 982 genes were curated from 21 DNA replication stress signatures and 894 were recovered in TCGA-PRAD. Univariate cox regression analysis identified 198 genes that were significantly related to PCa biochemical recurrence in the TCGA-PRAD datasets (Additional file 1: Table S6). The bootstrap approach further selected 136 of 198 prognostic genes that were robust to sample resampling and that were also identified in validating datasets (Additional file 1: Table S7). Furthermore, we adopted the Boruta algorithm and narrowed down the selected genes to 47 genes that were confirmed more relevant to recurrence (Additional file 3: Figure S2A, B). As shown in Fig. 2A, 47 genes were ranked according to the importance degree inferred by the Boruta algorithm, with the top 5 including EMD, HJURP, PLK1, TROAP, and CENPK. In addition, we confirmed that these 47 genes have higher mRNA expression in recurrent PCa samples in TCGA-PRAD (Additional file 3: Figure S2C).

### Construction of DNA replication stress signature

Using features selected by the Boruta algorithm, we benchmarked 7 survival-related machine learning algorithms including Enet, lasso, Ridge, XGBoost, plsRcox, SuperPC, and CoxBoost, to screen for a hyperparameter-tuned model with the best accuracy and lower risk of overfitting. Nested CV with outer 10 folds for validation and inner 5 folds for hyperparameter tuning was performed in the TCGA-PRAD. As shown in Fig. 2B–D and Additional file 1: Table S8, the XGBoost survival model achieved the best performances with the highest mean C-index (0.725), lowest mean IBS (0.156), and highest mean AUC values (1-year: 0.807; 3-year: 0.746; 5-year: 0.703: 10-year: 0.742). The XGBoost model with tuned hyperparameters was then fitted to the entire TCGA-PRAD dataset and referred as RSS. The inferred feature contribution to RSS was demonstrated in Fig. 2E, with the top 5 features including EMD, CCNE2, PTTG1, TROAP, and TK1.

### Evaluation of DNA replication stress signature

**Fig. 3:**
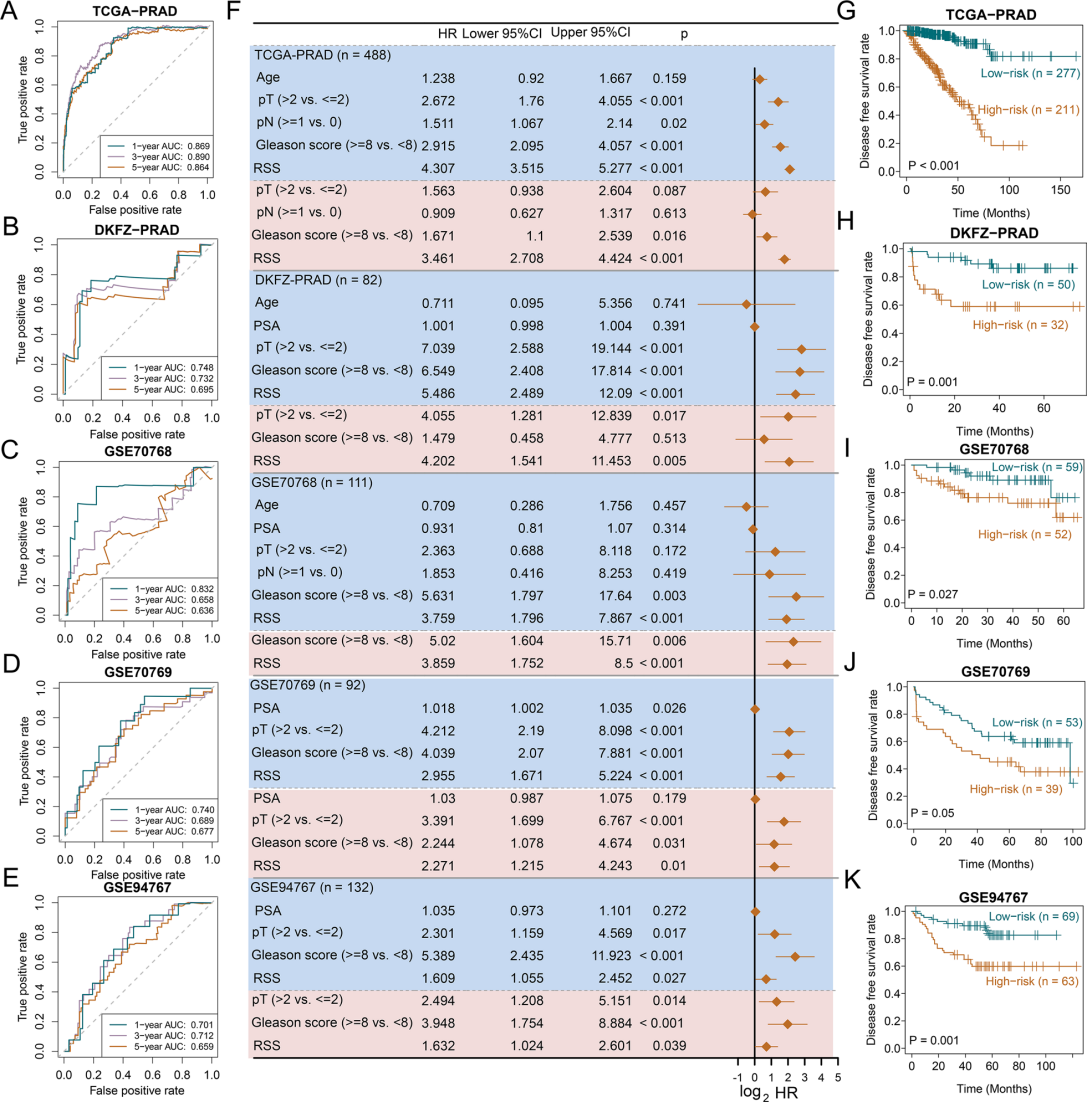
Evaluation of the DNA replication stress signature (RSS) in multiple cohorts. Time-dependent area under the receiver operating characteristic curve (AUC) at 1-, 3-, and 5-year in the **A** TCGA-PRAD, **B** DKFZ-PRAD, **C** GSE70768, **D** GSE70769, **E** GSE94767 datasets. **F** Forest plots demonstrate the hazard ratio (HR), 95% confidence interval (CI), and the corresponding P values of both univariate Cox regression analysis (shown in the pink shading area) and multivariate Cox regression analysis (shown in the blue shading area) in 5 prostate cancer cohorts. Kaplan–Meier plots of the **G** TCGA-PRAD, **H** DKFZ-PRAD, **I** GSE70768, **J** GSE70769, **K** GSE94767 datasets. High- and low-risk groups are determined by the universal cutoff of 0.536. P values are derived from log-rank test. PSA stands for prostate-specific antigen; pT refers to the pathological T stage; pN refers to the pathological N stage; RSS represents replication stress signature

Next, we interrogated the prognostic values of RSS in the TCGA-PRAD training cohort and 4 external validating cohorts using 1-year AUC, 3-year AUC, 5-year AUC, and C-index. The 1-year, 3-year and 5-year AUC values were 0.869, 0.890, 0.864 for TCGA-PRAD, and 0.748, 0.732, 0.695 for DKFZ-PRAD, and 0.832, 0.658, 0.636 for GSE70768, and 0.740, 0.689, 0.677 for GSE70769, and 0.701, 0.712, 0.659 for GSE94767 (Fig. 3A–E). The C-index values were 0.851 for TCGA-PRAD, 0.700 for DKFZ-PRAD, 0.724 for GSE70768, 0.654 for GSE70769, and 0.670 for GSE94767. Overall, RSS showed robust predictive power across validating datasets.

We then conducted univariate and multivariate Cox regression analyses and revealed that RSS as a continuous variable was significantly associated with a shorter time to biochemical recurrence in all datasets and was therefore considered an independent risk factor for PCa recurrence. (TCGA-PRAD: HR: 3.461, 95%CI: 2.708–4.424; DKFZ-PRAD: HR: 4.202, 95%CI: 1.541–11.453; GSE70768: HR: 3.859, 95%CI: 1.752–8.500; GSE70769: HR: 2.271, 95%CI: 1.215–4.243; GSE94767: HR: 1.632, 95%CI: 1.024–2.601, Fig. 3F). In addition, we tested whether a fixed RSS threshold could be used to classify all included PCa patients as high- or low-risk. To this end, we used “SurvivalROC” R package and found that a cutoff point of 0.536 in GSE94767 was able to divide all patients into high- and low-risk groups with significant differences in time to recurrence for all datasets, as shown by the Kaplan–Meier analyses (all log-rank P <  = 0.05, Fig. 3G–K). Taken together, RSS conferred great potential to facilitate discrete risk stratification for primary PCa.

### Comparison of RSS to clinical variables and published signatures

**Fig. 4:**
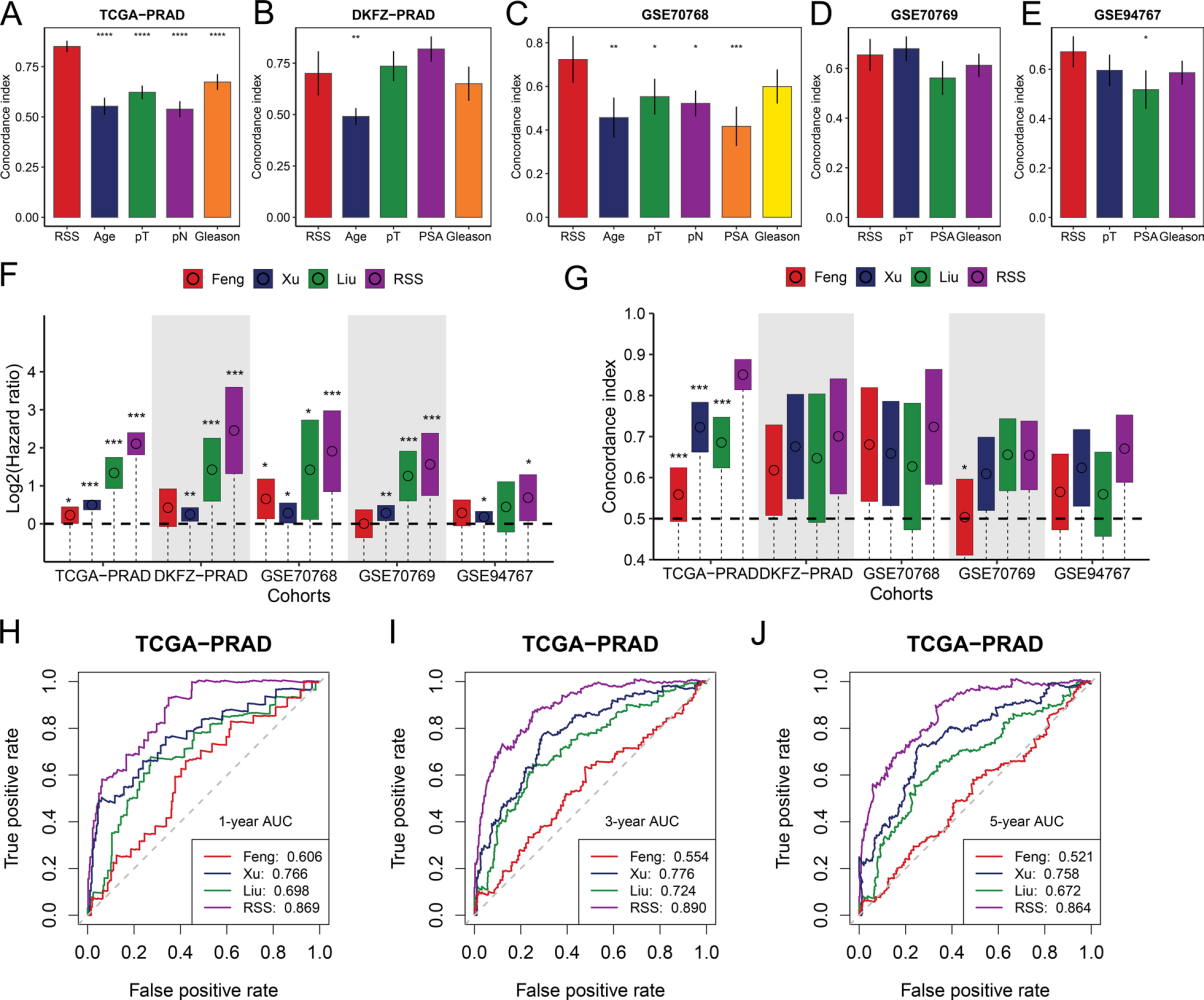
The predictive performance of replication stress signature (RSS) was compared with that of clinical features and prognostic signatures. Comparison of C-index between RSS and clinical features in the **A** TCGA-PRAD, **B** DKFZ-PRAD, **C** GSE70768, **D** GSE70769, **E** GSE94767 datasets. Data are presented as mean ± 95% confidence interval. **F** Univariate Cox regression analysis of prognostic signatures in 5 prostate cancer cohorts. Dots represent log2(hazard ratio). The upper and lower bounds of the bars indicate log2(95% confidence interval). **G** Comparison of C-index between RSS and other prognostic signatures across cohorts. Dots represent the mean C-index while the upper and lower bounds of the bars indicate a 95% confidence interval. Comparison of Time-dependent area under the receiver operating characteristic curve (AUC) among prognostic signatures at **H** 1-, **I** 3-, and **J** 5-years in the TCGA-PRAD dataset. The asterisks are used to denote the statistical P value (*P < 0.05; **P < 0.01; ***P < 0.001, ****P < 0.0001)

Since clinical variables such as Gleason score, serum PSA, and TNM staging are commonly used to guide PCa management and predict prognosis, we compared them with our proposed RSS using the C-index. Overall, RSS showed better predictive accuracy than most clinical features in the TCGA-PRAD and GSE70768 datasets, and non-inferior predictive power in the DKFZ-PRAD, GSE70769, and GSE94767 datasets (Fig. 4A–E).

We next compared RSS with published PCa signatures and found that RSS had a higher hazard ratio, C-index, and AUC values than other signatures in the TCGA-PRAD cohort (Fig. 4F–J) and demonstrated superior or comparable results in other cohorts (Additional file 4: Figure S3 and Additional file 1: Table S9).

### Multiomic comparison between RSS-high and RSS-low groups in TCGA-PRAD

**Fig. 5:**
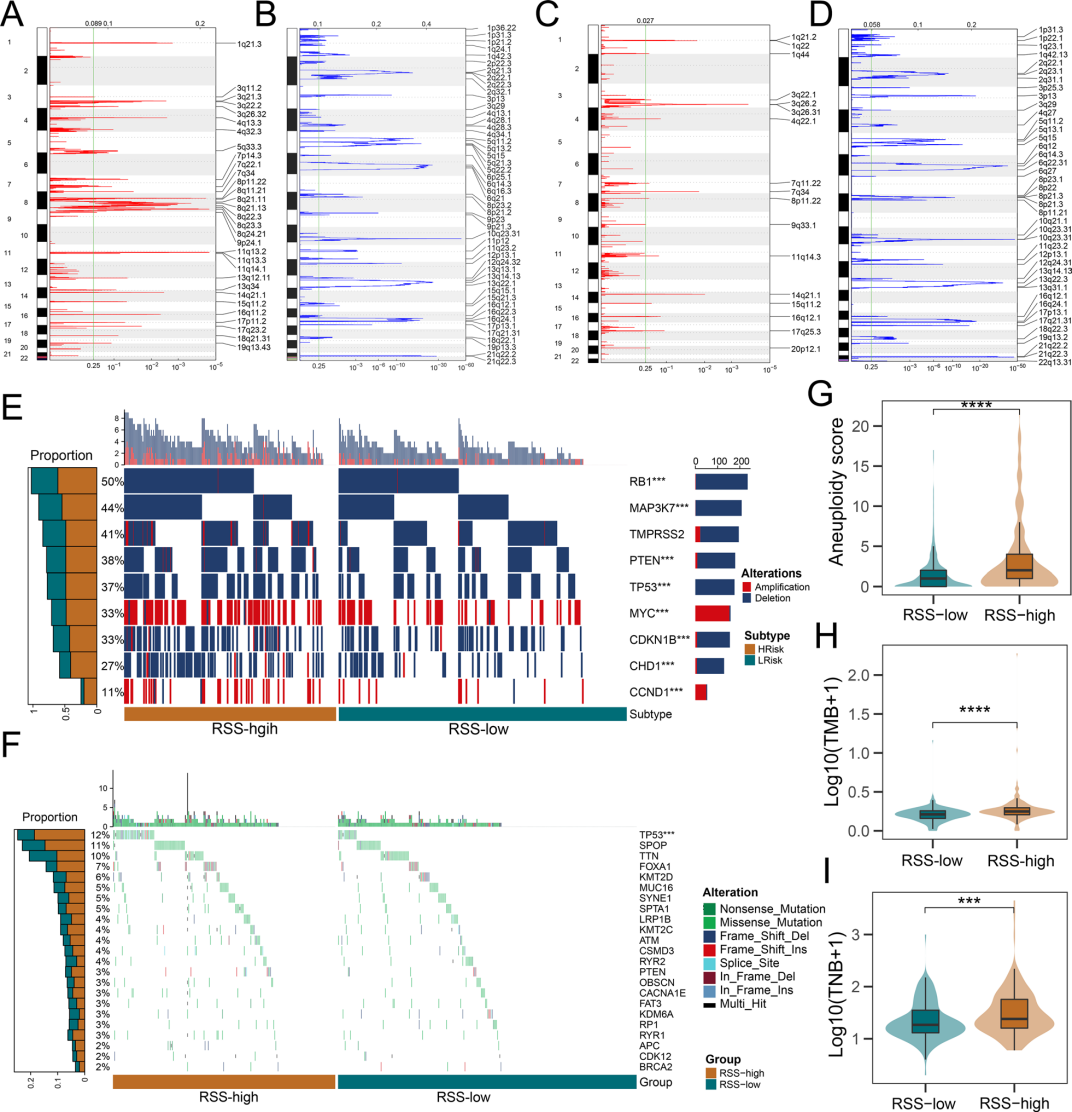
Multi-omic characterization of RSS-high and RSS-low patients. Recurrent copy number **A** amplification and **B** deletion regions detected in the RSS-high group. Recurrent copy number **C** amplification and **D** deletion regions detected in the RSS-low group. **E** The oncoprint of genes affected by recurrent copy number alterations. The bar plot on the right side of the oncoprint indicates the corresponding proportion of alterations in each group. **F** The oncoprint of common somatic gene mutations. The bar plot on the right side of the oncoprint indicates the corresponding proportion of somatic mutations in each group. The distribution of **G** aneuploidy score, **H** tumor mutation burden, and **I** tumor neoantigen burden between RSS-high and RSS-low patients in the TCGA-PRAD dataset. The upper and lower bounds of the boxes represented 75th and 25th percentiles while the center lines in the boxes indicate the median values. The asterisks denote the statistical P value (*P < 0.05; **P < 0.01; ***P < 0.001, ****P < 0.0001)

Using GISTIC2.0, we found that the RSS-high group conferred more recurrent copy number alterations than the RSS-low group (Fig. 5A–D). Common deletions in prostate cancers such as 8p21.3 (RSS-high: 70.4%; RSS-low: 60.2%) and 13q14.13 (RSS-high: 61.2%; RSS-low: 42.8%) occurred more frequently in the RSS-high group than the RSS-low group. Similarly, recurrent CNV gains such as 8q24.21 were only detected in the RSS-high group (46.4%). We also investigated common genes affected by somatic copy number alterations. As shown in Fig. 5E and Additional file 1: Table S10, RSS-high patients conferred more deletions of tumor suppressor genes such as TP53 (RSS-high: 48.5%; RSS-low: 29.0%), PTEN (RSS-high: 45.5%; RSS-low: 29.0%), and RB1(RSS-high: 60.5%; RSS-low: 41.2%; all adjusted P < 0.001). Also, amplification of MYC (RSS-high: 46.0%; RSS-low: 22.4%) and CCND1 (RSS-high: 18.5%; RSS-low: 4.4%) was frequently identified in the RSS-high group (all adjusted P < 0.001). We next compared common somatic mutations between RSS-high and RSS-low groups (Fig. 5F and Additional file 1: Table S11) and found that TP53 was more frequently mutated in RSS-high patients (18.6%) than in RSS-low patients (6.2%). Although DNA damage response-related gene mutations were rare (BRCA2: 2%; ATM: 4%) in primary PCa and comparable between groups, the RSS-high group had significantly higher HRD-detect scores (Additional file 5: Figure S4), suggesting that despite the low mutation rate, RSS-high patients exhibited more homologous deficiency at the transcriptome level. Additionally, the aneuploidy score, tumor mutation burden, and tumor neoantigen burden were significantly higher in the RSS-high group than in the RSS-low group (all P < 0.05, Fig. 5G–I). Altogether, the RSS-high group displayed a genomic pattern reminiscent of advanced PCa.

### Association of RSS with clinical features and biological processes

**Fig. 6:**
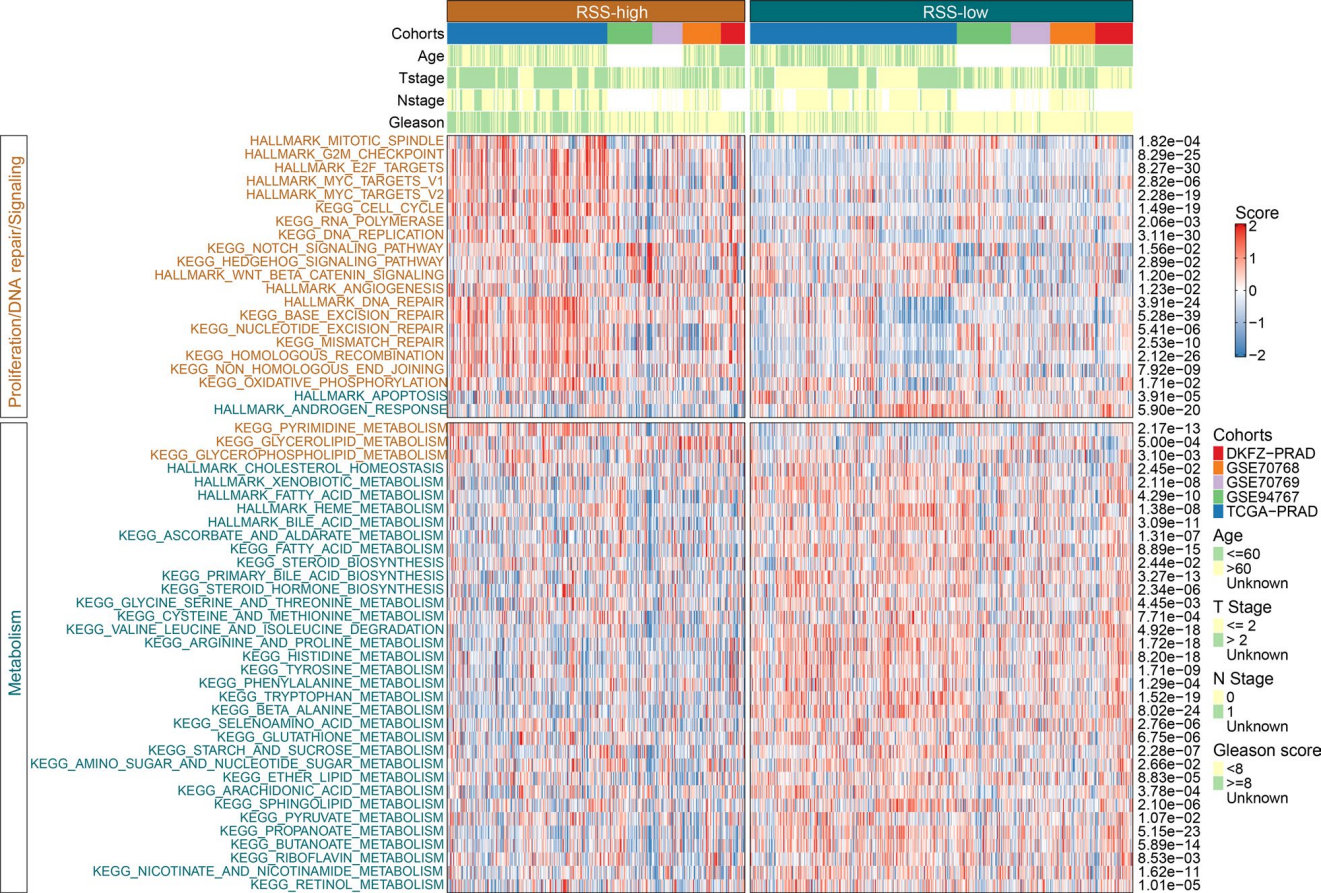
The associations of clinicopathologic and biological features with the replication stress signature. The upper panel of the heatmap showed the distribution of clinical characteristics between RSS-high and RSS-low patients. The lower panel demonstrated z-scores of single sample gene set enrichment analysis. The different colors of right-sided text annotation indicate the relative enrichment of pathways in the corresponding groups. The annotations on the left side indicate statistical P values

We compared the clinical characteristics between RSS-high and RSS-low groups in all cohorts (Additional file 1: Table S12). In general, RSS-high patients were associated with more advanced tumor stage (T3-4), and higher Gleason scores (> = 8). We next investigated the impact of RSS on biological pathways using ssGSEA. As shown in Fig. 6 and Additional file 1: Table S13, the RSS-high group was significantly enriched with cell-cycle related pathways such as mitotic spindle, E2F targets, G2M checkpoint, MYC targets, DNA replication, and DNA repair-related pathways such as base excision repair, nucleotide excision repair, mismatch repair, and several cancer-related pathways such as WNT/beta-catenin signaling, Notch signaling, and angiogenesis (all P < 0.05). In contrast, the RSS-low group was significantly associated with increased androgen response and apoptosis (all P < 0.05). Of note, a lower RSS score was characterized by significant activation of metabolism-related pathways such as fatty acid metabolism, steroid biosynthesis, and amino acid-related metabolism pathways while only several metabolism pathways such as oxidative phosphorylation and pyrimidine metabolism were enriched in the RSS-high group (all P < 0.05). In summary, the RSS-high group was highly proliferative and invasive, whereas the RSS-low group had elevated androgen response and metabolism activity that are reminiscent of prostate tumors derived from primary human luminal epithelial cells [31].

### Association of RSS with the immune microenvironment

**Fig. 7:**
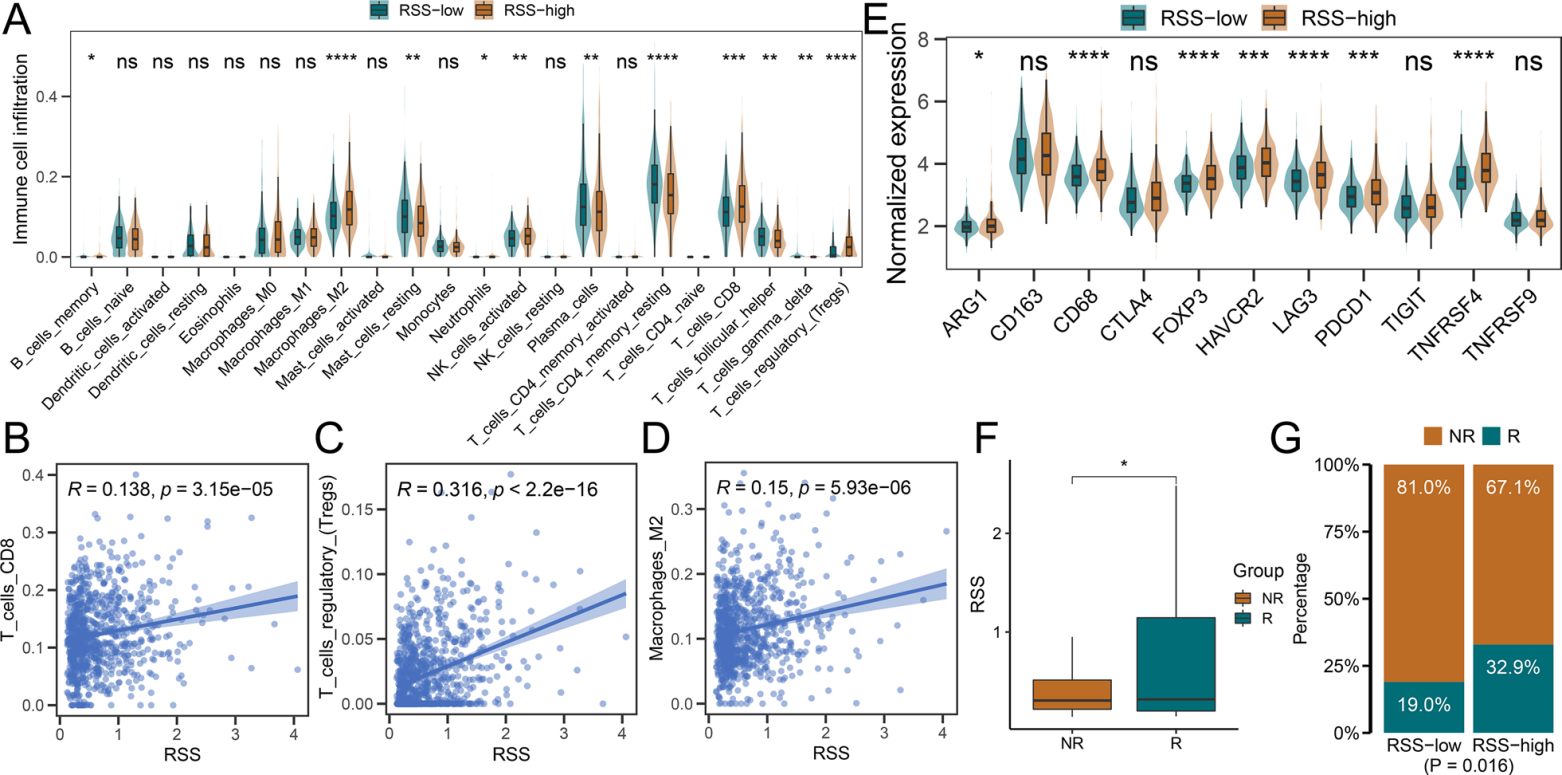
The association between replication stress signature and immune cell infiltrations in the Meta-cohort. **A** The result of CIBERSOR analysis. **B** The scatterplot between RSS and CD8 + T cells. **C** The scatterplot between RSS and regulatory T cells. **D** The scatterplot between RSS and M2 macrophages. The correlation coefficient *R* and corresponding *P* values are derived from Spearman’s rank correlation analysis. **E** The expression of immune-related genes in RSS-high and RSS-low patients. **F** The distribution of RSS between atezolizumab responders and non-responders. **G** The percentages of responders and non-responders in RSS-high and RSS-low groups. “R” represents responders while “NR” indicates non-responders in **F** and **G**. The upper and lower bounds of the boxes represented 75th and 25th percentiles while the center lines in the boxes indicate the median values. The asterisks represented the statistical P-value (*P < 0.05; **P < 0.01; ***P < 0.001, ****P < 0.0001)

Replication stress was reported to activate pro-inflammatory responses and change the tumor microenvironment [32, 33]. We, therefore, leveraged CIBERSORT to quantify immune cell infiltration in 905 PCa samples (Additional file 1: Table S14) and investigated the association between RSS and immune infiltration. Interestingly, the RSS-high group showed an increased proportion of CD8 + T cells, regulatory T cells, and M2 macrophages compared to the RSS-low group (all P < 0.001, Fig. 7A). Furthermore, RSS was positively correlated with proportions of CD8 T cells (R = 0.138, P < 0.001, Fig. 7B), regulatory T cells (R = 0.316, P < 0.001, Fig. 7C) and M2 macrophages (R = 0.15, P < 0.001, Fig. 7D). Additionally, the RSS-high group showed significantly higher expression of immunosuppressive markers such as FOXP3, HAVCR2, LAG3, PDCD1, and ARG1 (all P < 0.05, Fig. 7E). As higher RSS exhibited more of an immune-exhausted phenotype, we asked whether RSS could influence the therapeutic response to immune checkpoint inhibitors in cancer patients. We then calculated RSS scores for the IMvigor210 cohort and found that atezolizumab responders had significantly higher RSS scores than non-responders (P < 0.05, Fig. 7F). We also used the threshold 0.536 to stratify the cohort into RSS-high and RSS-low groups and found significantly more responders in RSS-high patients (32.9%, P = 0.016, Fig. 7G).

### In silico discovery of potential targets and drugs for RSS-high PCa patients

**Fig. 8:**
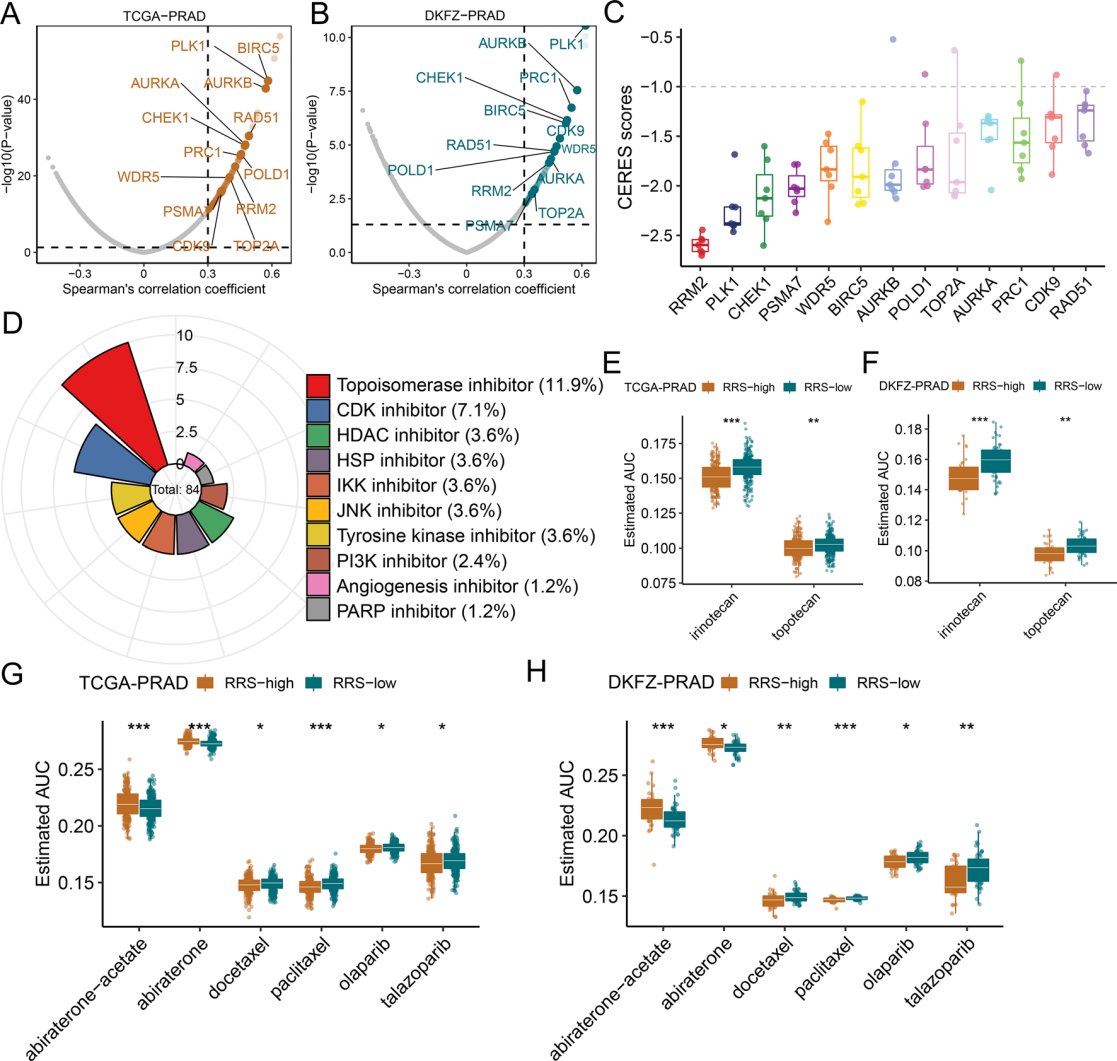
Identification of potential therapeutic targets and agents for RSS-high patients. Dot plots of the correlation coefficients derived from Spearman’s rank correlation analysis between RSS and druggable mRNA expression in the **A** TCGA-PRAD and **B** DKFZ-PRAD datasets. Light-colored dots represent potential targets that pass the threshold in Spearman’s rank correlation analysis (R > 0.3 and adjusted P < 0.05), while dark-colored dots indicate targets that were also selected by CERES analysis. **C** The distribution of CERES scores of identified targets in prostate cancer cell lines. **D** The composition of chemical compounds selected by CMap analysis. Only the top 10 drug categories are displayed. The inferred AUC values of irinotecan and topotecan were compared between RSS-high and RSS-low patients in the **E** TCGA-PRAD and **F** DKFZ-PRAD datasets. The inferred AUC values of ADT, taxanes, and PARP inhibitors were compared between RSS-high and RSS-low patients in the **G** TCGA-PRAD and **H** DKFZ-PRAD datasets. The upper and lower bounds of the boxes represented 75th and 25th percentiles while the center lines in the boxes indicate the median values. The asterisks represented the statistical P-value (*P < 0.05; **P < 0.01; ***P < 0.001, ****P < 0.0001)

To identify potential targets for RSS-high patients, we first performed Spearman’s rank correlation analysis between RSS and RNA expression of druggable genes in the TCGA-PRAD and DKFZ-PRAD cohorts (Fig. 8A, 8B and Additional file 1: Table S15, S16) and considered a common subset of positively correlated genes (R > 0.30, FDR < 0.05) in both cohorts as RSS-related targets (n = 54). In addition, we leveraged CERES scores to measure the essentiality of the RSS-related targets in 7 PCa cell lines and narrowed down to 13 potential therapeutic targets with CERES scores mostly < -1 (Fig. 8C). We found that many therapeutic targets such as TOP2A, CDK9, CHEK1, RRM2, and AURKB were tightly linked to cell cycle processes.

Next, CMap analysis was carried out to infer potentially effective chemical compounds. For this purpose, we performed differential gene analysis across 5 PCa cohorts and applied meta-analysis with a random effect model to make a consensus list of differentially expressed genes (Additional file 6: Figure S5 and Additional file 1: Table S17). The 150 most upregulated genes and 150 most downregulated genes were then used as the RSS signature to predict the CMap score for each chemical compound. Through this, we identified a total of 84 compounds with a CMap score below − 95 and with the ability to reverse RSS signature (Fig. 8D and Additional file 1: Table S18). Of 84 compounds, 11.9% and 7.1% belonged to topoisomerase inhibitors and CDK inhibitors, respectively. To improve the confidence of CMap inference, the PRISM-derived drug response data were used to infer AUC values of the compounds selected by CMap. We found that 2 topoisomerase inhibitors, including irinotecan and topotecan, consistently showed lower AUC values in the RSS-high groups in both TCGA-PRAD and DKFZ-PRAD cohorts (Fig. 8E, F), which supported our inference that topoisomerase was one of the potential targets. In addition, we investigated whether RSS could predict therapeutic response to conventional PCa therapy. As shown in Fig. 8G, H, RSS-high patients were more susceptible to taxane-based chemotherapy including docetaxel and paclitaxel, and PARP inhibitors including olaparib and talazoparib. In contrast, RSS-low patients were more sensitive to ADT such as abiraterone.

### Knockdown of FEN1 and RFC5 inhibits cell growth

**Fig. 9:**
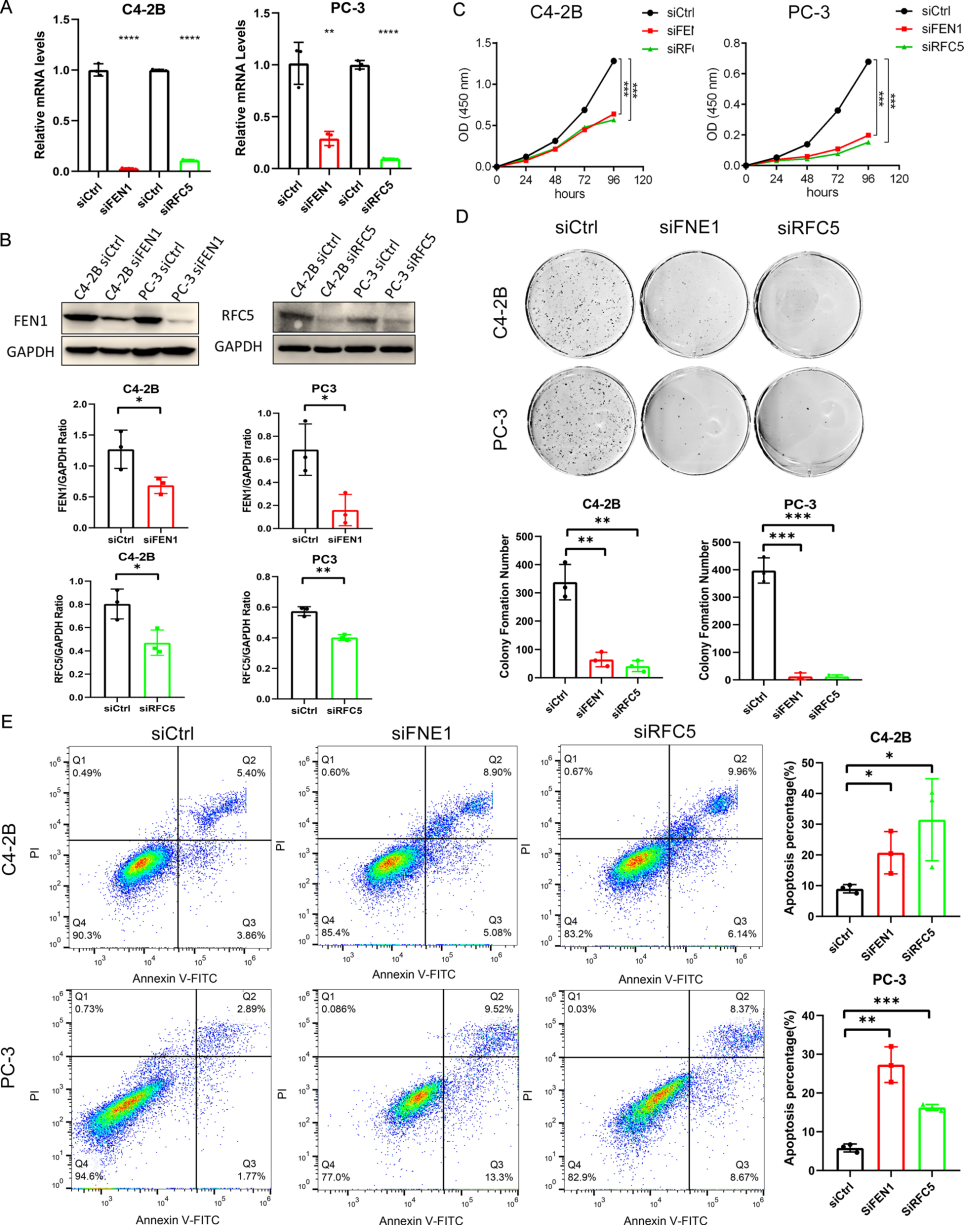
Knockdown of FEN1 and RFC5 inhibits cell growth and promotes apoptosis. Levels of FEN1 and RFC expression in C4-2B and PC-3 are decreased by siRNA knockdown as measured in the **A** real-time qPCR and **B** Western blot analysis. Comparison of cell growth among the control, FEN1, and RFC5 knockdown groups in C4-2B and PC-3 via **C** CCK-8 and **D** colony formation assays. **E** Measurement of cell apoptosis in control, FEN1, and RFC5 knockdown groups by flow cytometry. Cells are stained with Annexin V-fluorescein 5-isothiocyanate/PI assay. The asterisks represented the statistical P-value (*P < 0.05; **P < 0.01; ***P < 0.001, ****P < 0.0001)

We chose FEN1 and RFC5 for experimental validation because they showed higher mRNA expression in recurrent PCa and are rarely investigated in PCa. We first confirmed the successful knockdown of FEN1 and RFC5 at mRNA and protein levels in C4-2B and PC-3 cells(Fig. 9A, B and Additional file 7). We then performed CCK-8 and colony formation assays in transfected C4-2B and PC-3 cells, which revealed that the knockdown of FEN1 and RFC5 significantly inhibited cell growth (all P < 0.05, Fig. 9C, D). In addition, we used AV and PI staining to assess the percentages of apoptotic cells after transfection. Results showed increased cellular apoptosis rates in C4-2B and PC-3 cells after knocking down FEN1 and RFC5 (Fig. 9E). Taken together, FEN1 and RFC5 could promote PCa progression by promoting cell growth.

## Discussion

DNA replication stress, a major driver of genomic instability, is considered a cancer cell-specific vulnerability that is therapeutically exploitable in PCa [16, 32]. Given the high heterogeneity of PCa in patients [3, 4], a reliable method to assess replication stress in PCa samples is currently needed before targeting this tumor dependency. Herein, based on DNA replication stress-related signatures, we established a robust RSS to predict the degree of replication stress and clinical prognosis of primary PCa in 5 cohorts through benchmarking 7 machine learning algorithms. We showed that the knockdown of FEN1 and RFC5 inhibited cell growth in PC-3 and C4-2B. Additionally, we investigated whether RSS could help inform therapeutic decisions in primary PCa through in silico strategies and found that RSS-high patients were more responsive to immunotherapy. Also, 13 potential therapeutic targets (e.g. TOP2A, CDK9, and RRM2) and 2 therapeutic agents (i.e. irinotecan and topotecan) were identified for RSS-high patients. Overall, this study established a novel and robust RSS that reflects replication stress levels and predicts prognosis and therapeutic responses in PCa.

Machine learning approaches have been increasingly used to predict patient survival [34, 35]. However, it’s still a challenge to successfully apply such techniques in clinical practices. The methods used in this study are capable of handling heterogeneous and high-dimensional data and are easy to implement for survival analyses [36, 37]. Lasso, Ridge, and Enet belong to penalized regression-based methods which tackle problems of data multicollinearity and n <  < p, and mitigate model overfitting by applying regularization [36, 37]. Except Ridge, Lasso and Enet allow for variable selections by shrinking some of the coefficients toward 0 [38]. However, Lasso may fail to select the most relevant predictors when predictors are highly correlated with each other [38]. In contrast, Enet tends to give similar coefficients to highly correlated predictors [38]. CoxBoost and XGBoost are developed based on boosting methods that minimize prediction errors by combining a series of weak learners into a stronger learner. CoxBoost applies likelihood-based boosting to estimate the regression parameter vector beta and it can incorporate mandatory variables into the final model [23, 37]. XGBoost, proposed by Chen et al. in 2016 [24], is a highly effective and computationally efficient implementation of the gradient boosting algorithm that supports regularization and contains many tunable hyperparameters. This may help explain why XGBoost performed best in this study. SuperPC first selects genes that are related to an outcome of interest and then computes the first few principal components using these correlated genes [21]. The principle components are used in the regression model to predict survival. The interpretation of components is generally not straightforward because the number of genes driving the component could be large [36]. plsRcox implements partial least squares regression to fit Cox models [22]. Partial least squares is based on latent components that are a linear combination of all original predictors, making it difficult to interpret [36, 39]. As it employs all original predictors regardless of their relevance, its performance could be compromised [39], which may contribute to the low predictive power in the current study. Of note, the characteristics of training datasets could also have different effects on the accuracy of different machine-learning methods.

Our study suggested oncogenic roles of FEN1 and RFC5 in PCa cell lines. FEN1 encodes flap endonuclease 1 and is involved in DNA replication and repair [40]. It promoted cell proliferation and inhibited apoptosis in lung tumor cells and promoted hepatocellular carcinoma progression by USP7/MDM2-mediated P53 inactivation [40, 41]. A previous study also reported that higher FEN1 expression was positively correlated with higher Gleason scores [42]. RFC5 is a subunit of the replication factor complex that acts as a primer identification factor for DNA polymerase. It was found highly upregulated in multiple cancers [43–45], which was consistent with the results of our in vitro experiments. However, the underlying mechanisms of how FEN1 and RFC5 promote PCa progression require further investigation.

Our results suggested that RSS-high patients were more susceptible to immunotherapy. Previous studies reported that replication stress-inducing agents caused cytosolic DNA accumulation which, once sensed by pattern recognition receptors, could lead to STING activation [46, 47]. The STING signaling then promoted cellular senescence or elimination by the adaptive immune system [48]. Also, mice lacking STING expression were resistant to immune checkpoint inhibitors [49]. Therefore, patients with higher RSS may have activated STING signaling, which renders them more responsive to immune checkpoint inhibitors.

TOP2A encodes DNA topoisomerase II alpha, an enzyme involved in catalyzing double-stranded DNA breaking [50]. Overexpression of TOP2A was associated with a higher risk of PCa progression and metastasis, and was accompanied by higher TNM stages and Gleason scores [51]. It was found that ADT alone was insufficient to inhibit PCa cell survival in cases of TOP2A overexpression, which was also supported by our results that RSS-high group patients with higher TOP2A levels were less likely to benefit from ADT [52]. CDK9 encodes cyclin-dependent kinase 9, a member of the cyclin-dependent kinase family that regulates cell cycle and transcription [53]. Additionally, CDK9 promotes the overexpression of anti-apoptotic proteins such as MCL-1 and BCL-2 by phosphorylating RNA polymerase II [53]. siRNA Knockdown or pharmacological inhibitors of CDK9 in LNCaP cells significantly inhibited androgen receptor activity and caused cell apoptosis [53]. RRM2 is a subunit of ribonucleotide reductase that serves as a key enzyme for DNA synthesis by catalyzing de novo synthesis of deoxyribonucleotides [54]. Silencing RRM2 was reported to inhibit cell invasion and colony formation in PCa [55]. Taken together, many proposed therapeutic targets were experimentally proved to be oncogenic and druggable in PCa by previous studies, which were in line with our bioinformatic findings. Importantly, our study suggested that PCa patients with high RSS may benefit from targeting these genes.

This study developed a machine-learning framework for the construction of robust predictive models by benchmarking multiple survival models. The implementation of such a framework allowed us to establish RSS that showed superior predictive powers over most clinical features and signatures across multiple cohorts. Nevertheless, the lack of prospective cohort validation and functional validation of genes included in RSS is the major limitation of this study. Also, cohorts included in this study differed in terms of sample sizes, sequencing platforms, and clinical characteristics (Additional file 1: Table S19). In addition, cancer is increasingly viewed as an ecosystem in which tumor cells interact with immune and stromal cells. Therefore, drug sensitivity prediction based on cancer cell line data failed to appreciate the possible impact of the tumor microenvironment on cancer cells.

## Conclusion

Overall, this study established a stable and powerful model for predicting recurrence and therapeutic response in primary prostate cancer with the help of multiple machine-learning algorithms. The RSS model holds promise for prostate cancer risk stratification and therapeutic guidance.

## Supplementary Information

**Additional file 1: Table S1.** Gene sets of DNA replication stress. **Table S2.** Hyperparameters used in the machine learning algorithms. **Table S3.** Collection of prostate cancer signatures. **Table S4.** Gene lists of pathways. **Table S5**. The list of curated targetable genes. **Table S6.** The result of univariate Cox regression analysis of DNA replication stress-related genes. **Table S7.** The result of Bootstrapping-based Cox regression analysis. **Table S8.** The result of machine learning benchmark. **Table S9.** The result of signatures comparison. **Table S10.** Somatic copy number alterations in RSS-high and RSS-low groups. **Table S11.** Somatic mutation characteristics in the RSS-high and RSS-low groups. **Table S12.** Clinicopathologic characteristics of prostate cancers in the included cohorts. **Table S13.** The result of single sample gene set enrichment analysis in the Meta-cohort. **Table S14.** The CIBERSORT result of 905 prostate cancer samples. **Table S15.** The result of Spearman’s rank-order correlation between replication stress signature and druggable genes in the TCGA-PRAD cohort. **Table S16.** The result of Spearman’s rank-order correlation between replication stress signature and druggable genes in the DKFZ-PRAD cohort. **Table S17.** The result of a meta-analysis of differential gene analysis. **Table S18.** The result of CMap analysis. **Table S19.** Comparison of clinical characteristics among included cohorts.

**Additional file 2: Figure S1.** Identification and correction of batch effects among included cohorts. (A) The principal component analysis suggested significant batch effects in the included cohorts. (B) The principal component analysis indicated no obvious batch effect after correction.

**Additional file 3: Figure S2.** Results of univariate Cox regression analysis of genes selected by the Boruta algorithm. (A) Univariate Cox regression of 47 genes selected by the Boruta algorithm. Data are presented as log2(hazard ratio) ± log2(95% confidence interval). (B) Results of the Boruta algorithm iterations. Green indicates features considered important by the Boruta algorithm while blue represents shadow attributes.

**Additional file 4: Figure S3. **Comparison of signatures by time-dependent ROC analysis at 1, 3, and 5 years. Results were shown for (A) DKFZ-PRAD, (B) GSE70768, (C) GSE70769 and (D) GSE94767.

**Additional file 5: Figure S4.** The distribution of HRDetect-score between RSS-high and RSS-low patients.

**Additional file 6: Figure S5.**The volcano plot of meta-analysis of differential gene analysis. The dots represent the log2(Summary fold change) of available genes derived from a meta-analysis of differential gene analyses of the included 5 PCa cohorts. The horizontal lines of the corresponding dots are the 95% confidence intervals. Yellow dots represent genes with log2(Summary fold change) > 0.3 and adjusted P < 0.05, while dark dots denote genes with log2(Summary fold change) < -0.3 and adjusted P < 0.05.

**Additional file 7.** Unprocessed Western blot images.

## Data Availability

All data analyzed in this study are publicly available in TCGA, GEO, cBioPortal, CCLE, and PRISM Repurposing datasets. Essential scripts are available on the GitHub website (https://github.com/Sudolin/RSS).

## Abbreviations

PCa: Prostate cancer
ADT: Androgen deprivation therapy
PARP: Poly (ADP-ribose) polymerase
PSA: Prostate-specific antigen
DDR: DNA damage response
RSS: Replication stress signature
TCGA: The Cancer Genome Atlas
GEO: Gene Expression Omnibus
AUC: Area under the dose–response curve/area under the ROC curve
Enet: Elastic network
plsRcox: Partial least squares regression for Cox
XGBoost: EXtreme Gradient Boosting survival
SuperPC: Supervised principal component
CV: Cross-validation
C-index: Harrell’s concordance index
IBS: Integrated brier score
ssGSEA: Single-sample gene set enrichment analysis
CMap: Connectivity MAP
HR: Hazard ratio
CI: Confidence interval
ROC: Receiver operating characteristic curve
