# Supplementary figures and images for: A machine learning framework develops a DNA replication stress model for predicting clinical outcomes and therapeutic vulnerability in primary prostate cancer
Source: J Transl Med. 2023 Jan 12;21:20. doi: 10.1186/s12967-023-03872-7 (PMC9835390; doi:10.1186/s12967-023-03872-7)

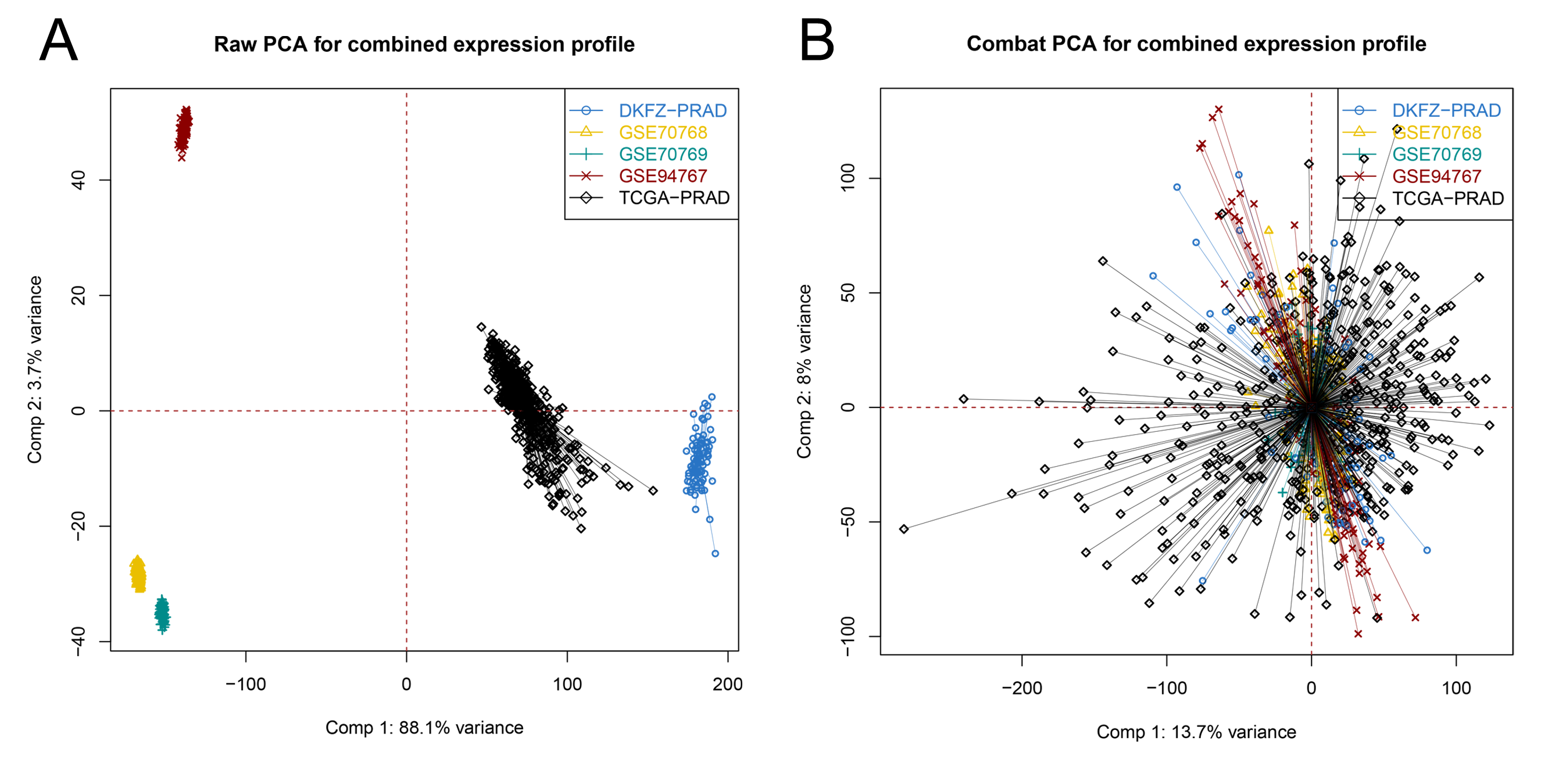

Supplement: Supplementary file 2 — Additional file 2: Figure S1. Identification and correction of batch effects among included cohorts. (A) The principal component analysis suggested significant batch effects in the included cohorts. (B) The principal component analysis indicated no obvious batch effect after correction. [file 12967_2023_3872_MOESM2_ESM.tif]

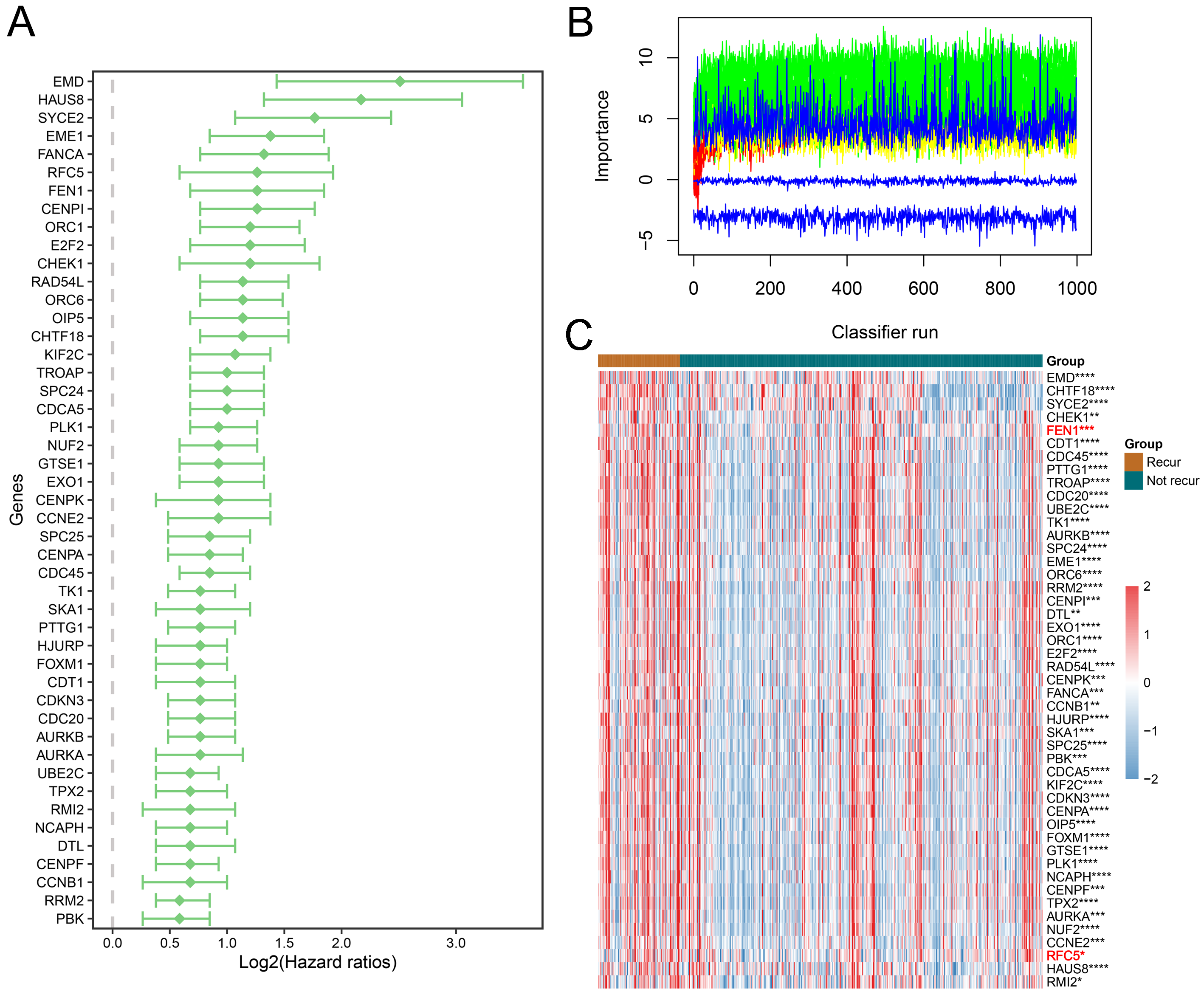

Supplement: Supplementary file 3 — Additional file 3: Figure S2. Results of univariate Cox regression analysis of genes selected by the Boruta algorithm. (A) Univariate Cox regression of 47 genes selected by the Boruta algorithm. Data are presented as log2(hazard ratio) ± log2(95% confidence interval). (B) Results of the Boruta algorithm iterations. Green indicates features considered important by the Boruta algorithm while blue represents shadow attributes. [file 12967_2023_3872_MOESM3_ESM.tif]

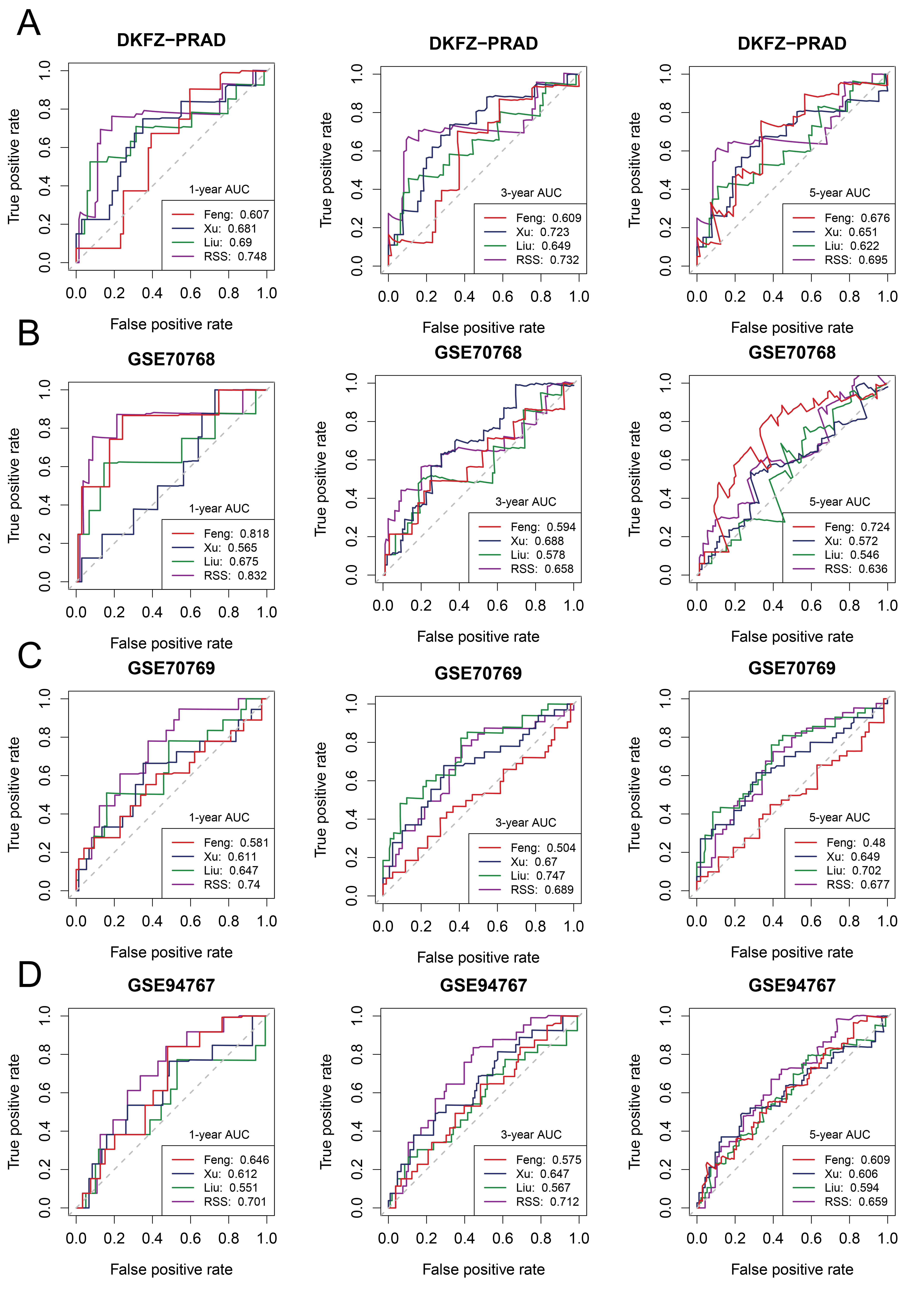

Supplement: Supplementary file 4 — Additional file 4: Figure S3. Comparison of signatures by time-dependent ROC analysis at 1, 3, and 5 years. Results were shown for (A) DKFZ-PRAD, (B) GSE70768, (C) GSE70769 and (D) GSE94767. [file 12967_2023_3872_MOESM4_ESM.tif]

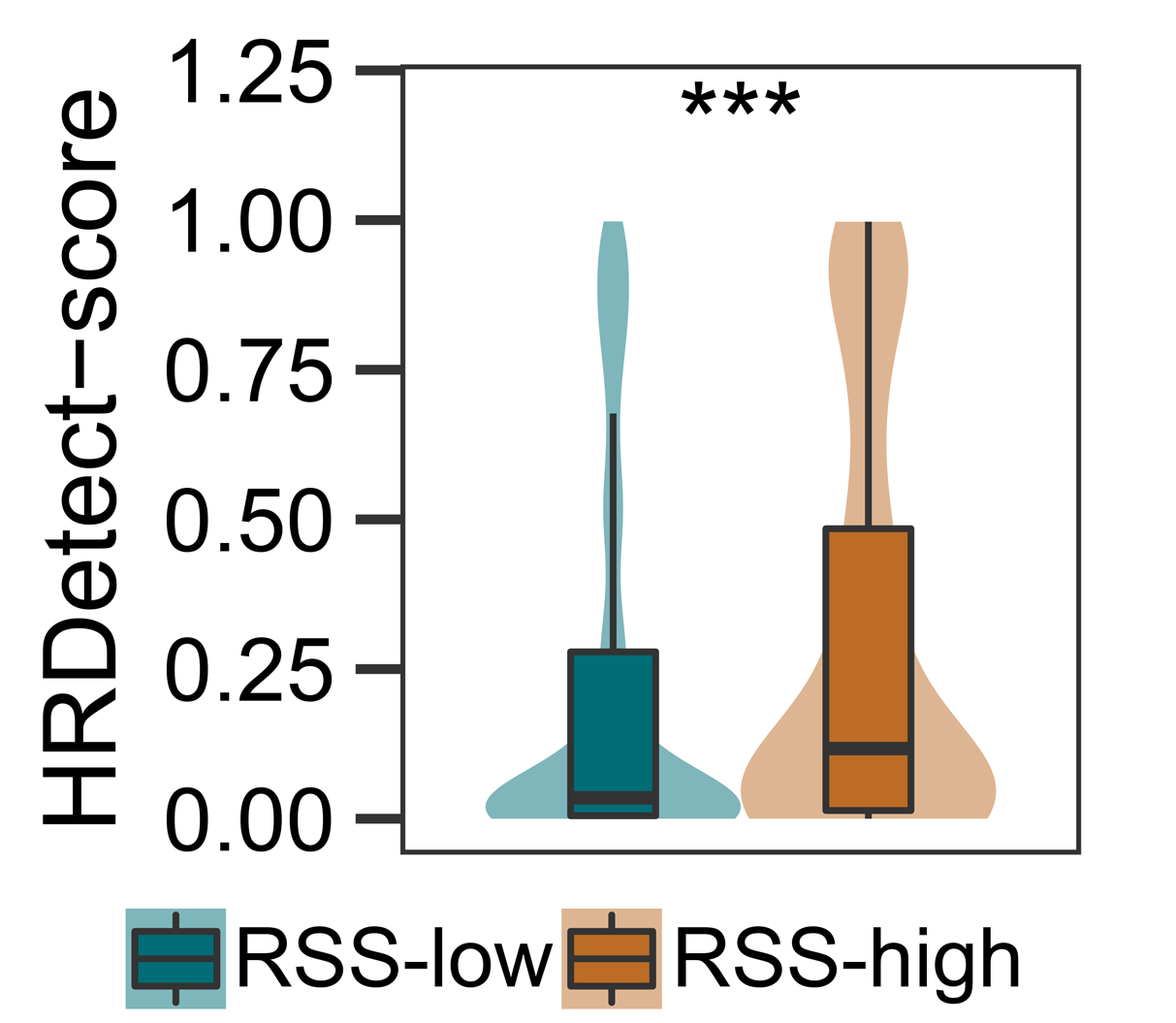

Supplement: Supplementary file 5 — Additional file 5: Figure S4. The distribution of HRDetect-score between RSS-high and RSS-low patients. [file 12967_2023_3872_MOESM5_ESM.tif]

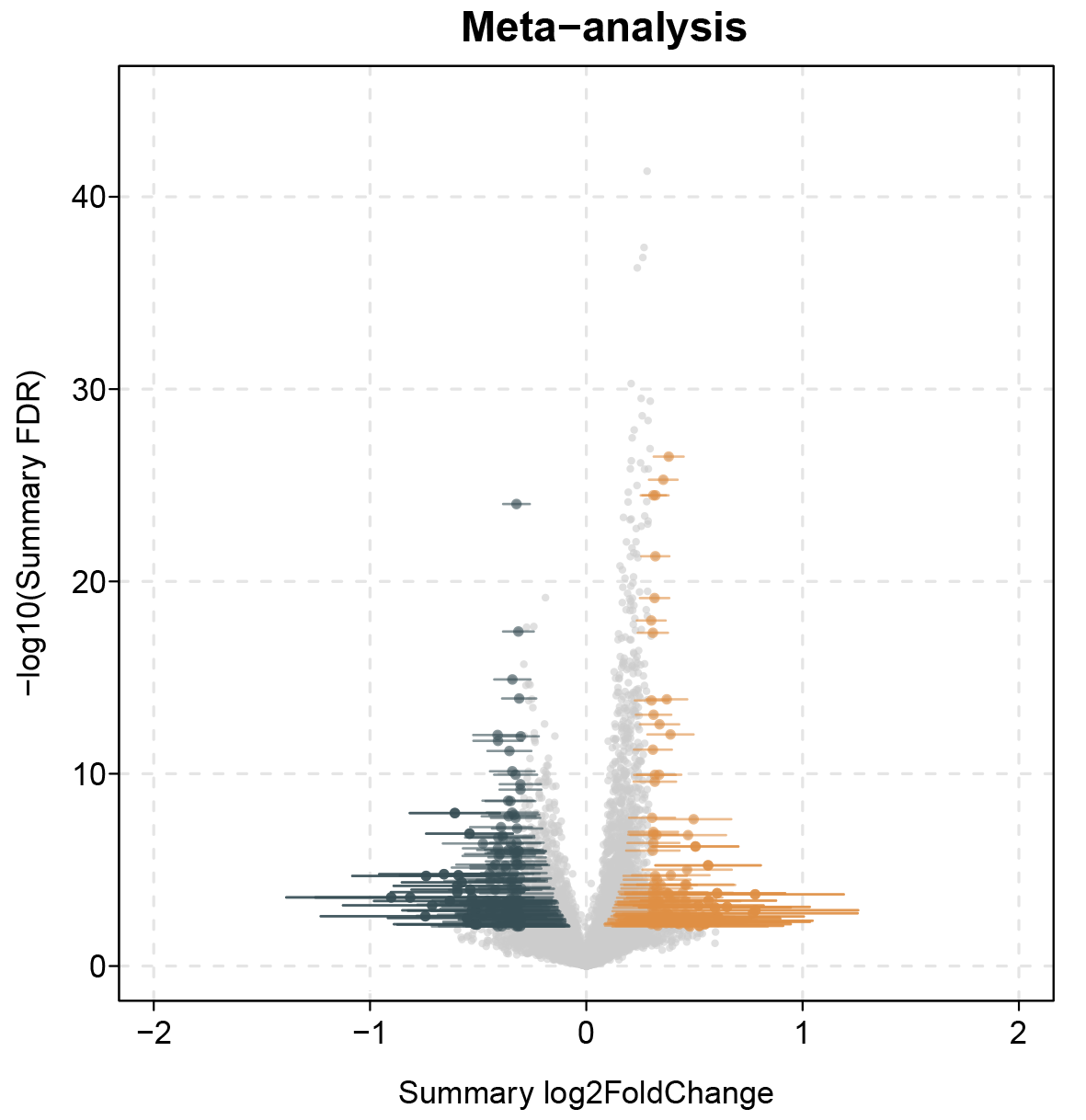

Supplement: Supplementary file 6 — Additional file 6: Figure S5.The volcano plot of meta-analysis of differential gene analysis. The dots represent the log2(Summary fold change) of available genes derived from a meta-analysis of differential gene analyses of the included 5 PCa cohorts. The horizontal lines of the corresponding dots are the 95% confidence intervals. Yellow dots represent genes with log2(Summary fold change) > 0.3 and adjusted P < 0.05, while dark dots denote genes with log2(Summary fold change) < -0.3 and adjusted P < 0.05. [file 12967_2023_3872_MOESM6_ESM.tif]
