# Supplementary material for: A machine learning framework develops a DNA replication stress model for predicting clinical outcomes and therapeutic vulnerability in primary prostate cancer
Source: J Transl Med. 2023 Jan 12;21:20. doi: 10.1186/s12967-023-03872-7 (PMC9835390; doi:10.1186/s12967-023-03872-7)
Supplement: Supplementary file 7 — Additional file 7. Unprocessed Western blot images. [file 12967_2023_3872_MOESM7_ESM.docx]

Unprocessed Western blot images.

RFC5

GAPDH

C4-2B siCtrl

C4-2B siRFC5

PC-3 siCtrl

PC-3 siRFC5

C4-2B siCtrl

C4-2B siRFC5

PC-3 siCtrl

PC-3 siRFC5

C4-2B siCtrl

C4-2B siRFC5

PC-3 siCtrl

PC-3 siRFC5


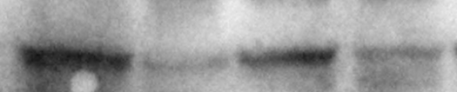

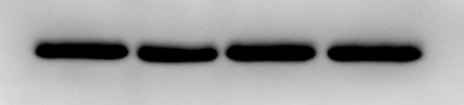

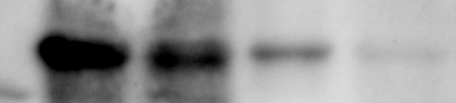

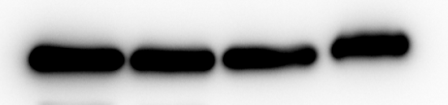

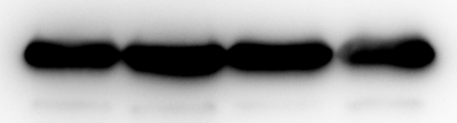

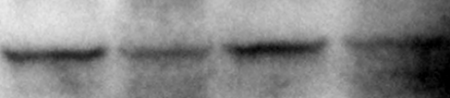

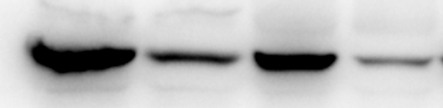

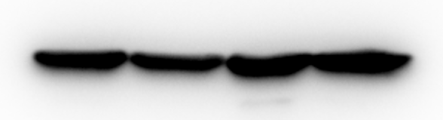

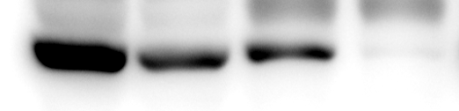

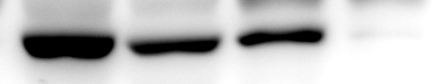

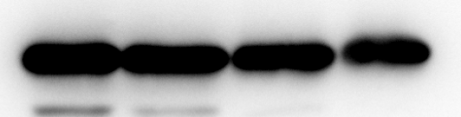

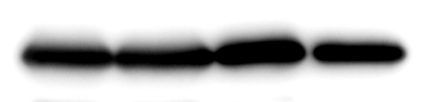


FEN1

GAPDH

C4-2B siCtrl

C4-2B siFEN1

PC-3 siCtrl

PC-3 siFEN1

C4-2B siCtrl

C4-2B siFEN1

PC-3 siCtrl

PC-3 siFEN1

C4-2B siCtrl

C4-2B siFEN1

PC-3 siCtrl

PC-3 siFEN1
